# Cullin-Conciliated Regulation of Plant Immune Responses: Implications for Sustainable Crop Protection

**DOI:** 10.3390/plants13212997

**Published:** 2024-10-26

**Authors:** Hongtao Wang, Zhiming Xie

**Affiliations:** 1 Laboratory of Biological Germplasm Resources Evaluation and Application in Changbai Mountain, School of Life Science, Tonghua Normal University, Yucai Road Tonghua 950, Tonghua 137000, China; hongtaowang@thnu.edu.cn; 2 College of Life Sciences, Baicheng Normal University, Baicheng 137000, China

**Keywords:** cullin, stress response, sustainable agriculture, cullin-mediated pathways, hormone signaling

## Abstract

Cullins are crucial components of the ubiquitin–proteasome system, playing pivotal roles in the regulation of protein metabolism. This review provides insight into the wide-ranging functions of cullins, particularly focusing on their impact on plant growth, development, and environmental stress responses. By modulating cullin-mediated protein mechanisms, researchers can fine-tune hormone-signaling networks to improve various agronomic traits, including plant architecture, flowering time, fruit development, and nutrient uptake. Furthermore, the targeted manipulation of cullins that are involved in hormone-signaling pathways, e.g., cytokinin, auxin, gibberellin, abscisic acids, and ethylene, can boost crop growth and development while increasing yield and enhancing stress tolerance. Furthermore, cullins also play important roles in plant defense mechanisms through regulating the defense-associated protein metabolism, thus boosting resistance to pathogens and pests. Additionally, this review highlights the potential of integrating cullin-based strategies with advanced biological tools, such as CRISPR/Cas9-mediated genome editing, genetic engineering, marker-associated selections, gene overexpression, and gene knockout, to achieve precise modifications for crop improvement and sustainable agriculture, with the promise of creating resilient, high-yielding, and environmentally friendly crop varieties.

## 1. Introduction

Cullins are an integral component of the E3 ubiquitin ligase complex, playing a critical role in the ubiquitin–proteasome pathway [1]. This pathway is essential for the targeted degradation of proteins, which is vital for maintaining cellular homeostasis. Cullins serve as scaffolds within the E3 ligase complex, facilitating the ubiquitination of specific substrate proteins. This ubiquitination marks the proteins for degradation by the proteasome, thus regulating various cellular processes by controlling protein levels [2,3].

Plants are continuously exposed to environmental stresses. They have internal defense mechanisms to combat these stresses, which vary from species to species. Thus, plant researchers mainly focus on translating and transforming information from well-known plants for application to other crucial food crops. Several indigenous proteins play important roles in maintaining the growth and development of plants under environmental stresses [4,5]. Among these, cullins also have a key role in plant growth and development regulation, including cell cycle regulation, DNA replication, the light response, circadian rhythm maintenance, photomorphogenesis, growth, and, primarily, protein degradation [2,6]. The cullin protein family appears highly expressed and can be found around the nucleus and cytosol.

**Figure 1:**
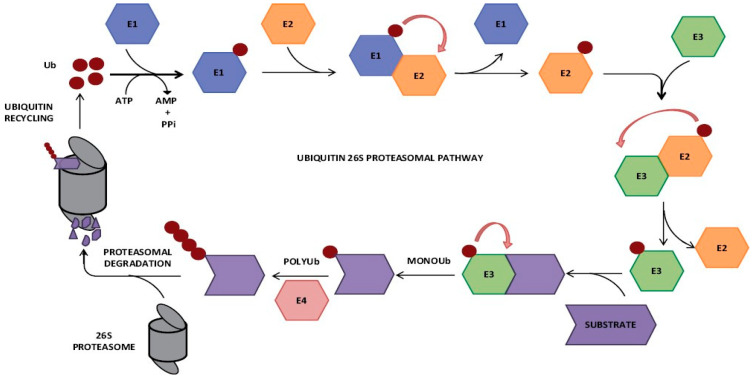
The ubiquitin–proteasome pathway. Free ubiquitin is activated and then transferred to a substrate protein by the action of three enzymes: E1, E2, and E3. There follow sequential rounds of ubiquitination, and the polyubiquitinated target protein is recruited to the 26S proteasome for proteolytic degradation, with recycling of free ubiquitin molecules. With permission from [19].

Cullin plays a crucial role in ubiquitination, which is an important mechanism in abscisic acid (ABA) signaling for protein stability and biological activation, as well as for translocation across the inner membrane [3,7]. This process modulates principal hormone agents that are involved in the synthesis and pathway of ABA. In turn, this modulation impacts plant responses to ABA. One of the most challenging aspects of decoding the procedure of ABA signaling is identifying ABA receptors. The ubiquitination process typically requires the cooperation of three enzymes: the E1 ubiquitin-activating enzyme that forms a thioester linkage with ubiquitin, the E2 ubiquitin-conjugating enzyme, and the E3 ubiquitin ligases, which recognize target proteins [7]. The E3 ubiquitin ligases are the principal family regulating numerous biological processes through 26S proteasome-assisted protein degradation [8]. This fact underscores the sophisticated role of cullin-family proteins and the ubiquitin–proteasome system in controlling fundamental aspects of plant evolution, growth, and stress responses [9,10,11]. Additionally, the ubiquitination process appears to be a key player in ABA signaling, affecting protein stability, translocation, and downstream gene expression in response to environmental stresses [12,13]. It has been revealed that ABA stimulus sensors and signaling proteomes undergo ubiquitination, highlighting their importance in the ABA-signaling process. ABI3-interacting protein 2 (AIP2), which functions as a RING-type E3 ubiquitin ligase for Abscisic Acid Insensitive 3 (ABI3), may be involved in the regulation of downstream genes in the ABA-signaling pathway [14]. AIP2 carries ubiquitination to ABI3 [15]. This regulation affects the production and transport of ABA, influencing the plant’s response by regulating key processes, such as abscisic acid synthesis and signal transduction [16]. The ubiquitin-26S proteasome can be found in cullins, which play a leading role in most hormone responses [10,11]. The ubiquitin-26S proteasome serves as a control point before, during, and after hormone synthesis, perception, and signaling, especially for reactions to auxin, jasmonic acid, gibberellins, ethylene, and abscisic acid [11,17]. Despite considerable advancements, a significant research gap remains in understanding the specific mechanisms governing cullin protein functions based on their subcellular localization and tissue-specific activities. A brief overview of the ubiquitin-proteasome pathway is given in Figure 1.

Understanding the complexity of cullin-mediated activities holds great potential for advanced agriculture, where leveraging these molecular regulators can enhance crop efficiency, resistance, and sustainability. Investigating the complex mechanisms governed by cullin-family proteins reveals a promising frontier in agricultural research, offering novel insights and transformative solutions for global food security challenges. A major knowledge gap regarding these enzymes in plants lies in the identification of their specific substrates. Additionally, the regulatory mechanisms governing cullin-based E3 ligases in plants remain poorly understood. In yeasts and metazoans, certain substrates are degraded by two or even three E3 ligases, and some E3 ligase components are targeted themselves for degradation by other classes of E3 ligases [18]. Thus, our understanding of degradation signaling in plants remains fundamentally poor. The ubiquitin/26S proteasome pathway has emerged as a central regulatory mechanism in eukaryotes, including plants, highlighting the importance of understanding cullin-mediated processes in agricultural contexts. However, gaps persist in our understanding of E3 ligases, particularly in regard to how cullin-based E3 ligases are regulated in plants and the specific substrates that they recognize.

In this review, we first discuss the basic roles of cullins in the ubiquitin–proteasome pathway, highlighting their significance in protein degradation and cellular homeostasis. We then explore the complexity of cullin proteins, detailing their structural characteristics and functional diversity. We examine the impact of cullins on plant growth and stress responses, highlighting their contribution to hormonal-signaling pathways; for example, those of abscisic acid and auxin. In the following sections, we discuss advanced research techniques, including high-throughput screening and structural biology, to illuminate the cullin interactions and substrate recognition. We also discuss the application of genome-wide analysis to understand the evolutionary and functional diversity of cullin gene families across plant species. Finally, we address the ethical and regulatory considerations associated with the distribution of cullin-based technologies in agriculture, emphasizing the importance of responsible innovation for viable crop advancement. 

## 2. Cullin Protein Complexity

**Figure 2:**
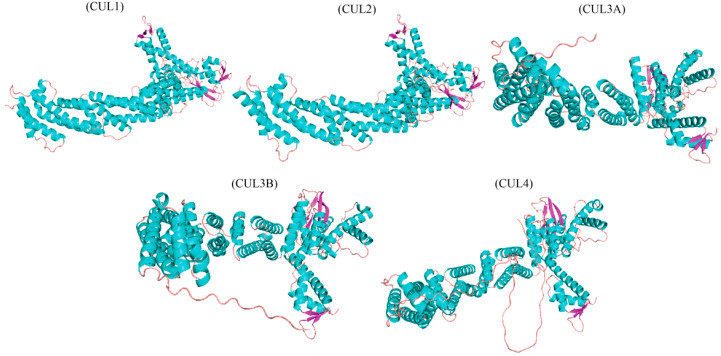
Structural comparison of different cullin proteins derived from PDB data. The 3D structures of CUL1, CUL2, CUL3A, CUL3B, and CUL4 are shown. The structures were visualized to highlight the distinct domain architectures and potential functional differences among the cullin family members in plant systems.

Cullin proteins form complex structures to perform their diverse functions. The RING (Radically Interacting with New Genes)-type E3 ligases play a crucial role in ubiquitination by simultaneously binding protein substrates and connecting E2 ubiquitination conjugates via the RING domains of E2 and E3 [20,21,22,23]. Cullins act as architectural units that assemble the major class of RING E3 ligases, represented by cullin–RING ligase complexes (CRLs) [24]. The 3D characteristics of cullins can be inferred from high-resolution structural analyses and biochemical experiments dedicated to CRLs. The configuration of cullins (and CRLs) in plants, deciphered via the molecular interaction between cullins and several CRL components, indicates the structural integrity and operational mechanisms of cullins [20]. The primary results obtained from such biochemical analysis serve as a framework for analogues found in other organisms, leading to the continued confirmation of the cullin protein across diverse species. Based on their structural similarities and differences, cullin proteins are categorized into different sub-members, ranging from CUL1 to CUL5, reviewed in [25]. These members are characterized by a twig-like terminal amino domain (NTD) with three cullin repeats (CR1 to CR3) [26]. The carboxyl-terminal domain (CTD) is globular, featuring the signature cullin homology domain (CH) and a conserved sequence of approximately 200 peptides. The D (UbV)-type cullin CTD joins with ROC1 or ROC2 (also known as RING-box protein; Rbx1 and Rbx2), ensuring the recruitment of the E2 enzyme loaded with ubiquitin for the ubiquitination mechanism. CUL1-ROC1 collaboration depends on multiple interactions, including CUL1’s α/β domain and the amino-terminal β-strand of ROC1 [27,28,29]. These interactions form a hydrophobic core between the two subunits, making CUL1-ROC1 indivisible. The cullin–RING motif is the core of CRLs, defining and distinguishing them structurally. Additionally, CUL1 also interacts with other proteins such as the S-phase kinase-associated protein 1 (Skp1)–CUL1-F-box (SCF) complex, and the CUL4A-RING–cullin-4 complex (CRL4A) shows that cullins are, indeed, the architectural factors in CRLs [30,31]. CRLs are built from two essential modules: (I) a substrate-identifying protein and a linker protein linked to the cullin via interactions with themselves and the RING domain that recruits an E2 ubiquitin-conjugating enzyme protein [32]. The SCF complexes comprise an F-box system with a common 40-peptide-long F-box motif at the C-terminus and an adaptor protein expressed from the *Skp1* gene, the latter of which interacts with F-box proteins and stabilizes their binding [33]. In contrast, the substrate-targeting units in the CRL4A consist of the adaptor protein DDA1 (DNA-damage-specific protein 1) and a family of DDB1 and CUL4-associated factors (DCAF) that are responsible for substrate recognition [34]. A structural comparison of different cullin proteins was derived from PDB data. The 3D structures of CUL1, CUL2, CUL3A, CUL3B, and CUL4 are shown in Figure 2. In CRL4A, the RL3 complex does not require a distinct connector part and uses the bifunctional molecule BTB (bric-a-brac, tramtrack, and broad complex), which directly binds to CUL3 while targeting the substrate [9]. Adaptors, such as Skp1 within SCF E3, Elongin C (EloC) in CRL2, CRL3 and CRL5, and BTB in RL3, have different overall structural folds. However, the linker conformations of SCF, CRL2, CRL3, and CRL5 E3s, named Skp1/BTB/Pox viruses and the zinc finger (POZ), share a common theme that contributes to their interaction with the amino terminus of cullin [35,36].

The roles of *Cul1* and *Cul3* in cell cycle regulation before embryogenesis have been demonstrated through Arabidopsis knockout experiments [37]. In Arabidopsis, the *AXR6* gene encodes CUL1, a key component of the SCF (Skp1-Cullin-F-box) E3 ubiquitin ligase complex, which plays a crucial role in the regulation of auxin-signaling pathways. This complex mediates targeted protein degradation, an essential mechanism for controlling various developmental processes, including embryogenesis. The involvement of CUL1 in SCF E3 ligase activity underscores its importance in auxin regulation, which is fundamental to plant growth and development [38]. Mechanistically, CUL1-based SCF controls plant cell proliferation by employing the Skp2 F-box protein, which regulates the ubiquitin-mediated degradation of p27 and p21 (cyclin-dependent kinase inhibitors), ultimately activating cyclin-dependent kinases [39]. CUL4-based ligases play critical roles in various biological processes, including DNA replication, chromatin remodeling, and the regulation of developmental processes [40].

**Figure 3:**
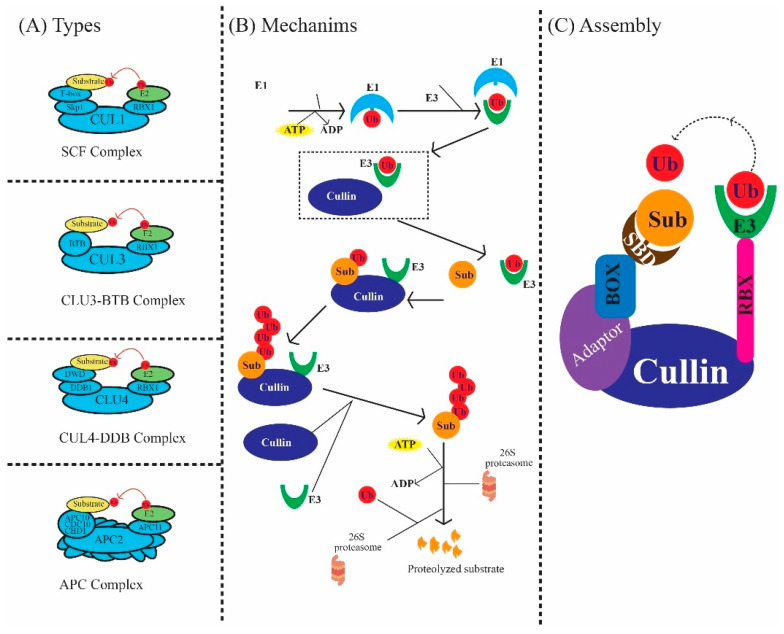
Illustration of the complexity of cullin proteins. (**A**) shows different types of protein complexes, including the SCF complex, CLU3–BTB complex, CLU4–DDB complex, and APC complex. (**B**) shows cullin-mediated protein degradation through ubiquitination. (**C**) shows the assembly of cullin along with different subunits.

Cullin proteins form the backbone of various cullin–RING E3 ubiquitin ligase complexes, each characterized by its specific cullin and adaptor proteins. The SCF complex, involving CUL1, partners with F-box proteins such as Skp1 to select and ubiquitinate substrates that are crucial for numerous cellular functions, including hormone signaling and environmental responses in plants [20,21,23]. The CUL3–BTB complex, with CUL3 and BTB domain-containing proteins, plays a key role in regulating plant development and stress responses by targeting specific proteins for degradation [28,41]. The CUL4–DDB complex, where CUL4 interacts with DDB1, is essential for DNA repair and maintaining genomic stability, highlighting its importance in protecting plant cells from genetic damage. Finally, the APC complex, another cullin-containing assembly, governs the cell cycle by controlling the degradation of regulatory proteins, ensuring proper progression through mitosis and meiosis in plant cells [30]. Reviewed in [31], as indicated in Figure 3A.

The CRL complexes operate through a carefully orchestrated mechanism to ubiquitinate target proteins, marking them for degradation [32]. The process begins with the activation of ubiquitin by the E1 enzyme, which uses ATP to form a high-energy bond with ubiquitin. This activated ubiquitin is then transferred to an E2 enzyme, which acts as a carrier, bringing ubiquitin to the CRL complex [30,31]. Within the CRL, the cullin protein, along with Rbx1 and specific adaptors, facilitates the transfer of ubiquitin from the E2 enzyme to the substrate [34]. The substrate is selected based on its interaction with adaptor proteins such as Skp1, BTB, or DDB1, ensuring specificity in ubiquitination. Multiple ubiquitin molecules are added to the substrate, forming a polyubiquitin chain that signals the 26S proteasome to degrade the tagged protein. This tightly regulated process is vital for maintaining cellular homeostasis and modulating various physiological processes in plants, as shown in Figure 3B [9].

The assembly of CRL complexes is a highly organized process that ensures the correct formation and function of these crucial protein complexes [37]. At the core of the assembly is the cullin protein, which serves as a scaffold to anchor the Rbx1 and the adaptor proteins, such as Skp1, BTB, or DDB1 [35]. These adaptors play a pivotal role in recognizing and recruiting specific substrate receptors, which determine the targets for ubiquitination. Once the substrate is bound, the ubiquitin molecule, carried by the E2 enzyme, is transferred to the substrate with the help of the E3 ligase activity, primarily mediated by Rbx1 [26], Figure 3C. This assembly ensures that only specific proteins are ubiquitinated and subsequently degraded by the proteasome, allowing the plant cell to finely tune its protein composition in response to developmental cues and environmental conditions. Understanding this assembly process is crucial for manipulating CRL activity in plant biotechnology applications, potentially leading to enhanced crop traits [39].

## 3. Molecular Mechanism

### 3.1. Salt Stress Response

In response to salt stress, E3 ligases, particularly those involving cullins, play a pivotal role in regulating protein stability and signaling pathways that are crucial for plant adaptation [42]. The E3 ligase RGLG1, which contains cullin components, is central to the ubiquitination process in the ABA-signaling pathway [43]. This pathway is activated under salt stress to induce stomatal closure, a key defense mechanism that helps prevent water loss by minimizing transpiration. By tagging specific proteins for degradation, RGLG1 ensures that the ABA-signaling pathway functions efficiently, facilitating a plant’s ability to respond to salt-induced stress conditions [43,44]. Additionally, the SOS (Salt Overly Sensitive) pathway, which involves the interplay of proteins such as SOS1, SOS2, and SOS3, works alongside E3 ligases to maintain ion homeostasis [45]. Here, ubiquitination processes help regulate the degradation of proteins that are not needed, thus fine-tuning the cellular response to elevated sodium levels and ensuring the removal of excess sodium ions via the Na⁺/H⁺ antiporter SOS1 [46]. This precise regulation of protein degradation and turnover is crucial for adapting to the osmotic stress caused by high salinity.

### 3.2. Heat Stress Response

E3 ligases, particularly those that involve cullin proteins, play a crucial role in a plant’s response to heat stress. At high temperatures, protein denaturation and misfolding pose significant threats to cellular functions [47]. E3 ligases tag these damaged proteins for degradation via the ubiquitin–proteasome system, ensuring that they do not accumulate to toxic levels [9,48]. This proteolytic pathway is vital for maintaining protein quality control and cellular homeostasis under thermal stress. Additionally, heat-shock factors (HSFs) and their downstream effectors, heat-shock proteins (HSPs), are tightly regulated by ubiquitination processes that are mediated by E3 ligases [49]. The modification of histone acetyltransferases (OsHATs) through ubiquitination can alter chromatin accessibility and the transcriptional activation of heat-responsive genes, highlighting the critical role of E3 ligases in modulating gene expression and facilitating plants’ adaptation to elevated temperatures [50].

### 3.3. Cold Stress Response

In cold stress conditions, E3 ligases with cullin complexes play a vital role in regulating a plant’s adaptive responses. They regulate the stability of key transcription factors such as ICE1 (Inducer of CBF Expression 1) and CBFs (C-repeat Binding Factors), which activate the expression of cold-responsive genes [51,52]. The MAPK-signaling cascade, which amplifies stress signals to activate ICE1 and CBFs, also interacts with ubiquitination pathways, where E3 ligases ensure that only necessary signaling components are retained [43,53]. This selective protein degradation is essential for fine-tuning the cellular response and ensuring the expression of genes that enhance cold tolerance. Moreover, E3 ligases modulate other transcription factors such as MYB15, adjusting their activity through phosphorylation and ubiquitination to optimize the expression of cold-responsive genes [52,54].

### 3.4. Interconnection of Pathways

E3 ligases, particularly those involving cullins, play key roles in various stress-response pathways, highlighting their importance in plant adaptability to environmental challenges. ABA signaling, a common part of stress responses, is intricately linked with E3 ligase activity, as these enzymes regulate the abundance and activity of key signaling molecules [7,13]. Negative regulators such as Protein Phosphatase 2Cs (PP2Cs) are also targeted for ubiquitination, allowing for sustained activation of ABA-signaling pathways [55]. The association and functioning of the CRL complex are regulated by a small ubiquitin-related modifier protein, RUB, also known as Nedd8. This domain is highly conserved among eukaryotes [22]. RUB is conjugated with the cullin component of the CRL complex with the help of an enzymatic process that is similar to the ubiquitination pathway. This complex, RUB-attaching enzyme 1 (RCE1), acts like an E2 ligase enzyme [56], while in Arabidopsis, two other proteins, AXR1 (auxin-resistant 1) and ECR1, form a heterodimer acting like an E1 enzyme [37]. Similarly, the RING-finger protein RBX1, the main component of the SCF complex, acts like E3 in the RUB conjunction pathway [57,58]. The RUB conjunction (Rubylation) pathway was first discovered in Arabidopsis through molecular selection for an auxin-resistant mutant, which is essential for ensuring the optimum functioning of the SCF complex to ensure its proper activity and regulation [59,60].

In the SCF, CUL3-BTB, and CUL4-DDB CRLs, RUB modification greatly impacts the assembly, disassembly, and regulation of CRL E3 complexes [61]. These CRL complex systems actively maintain cellular homeostasis by controlling the specificity of cullin rubylation, with the functionality of CRL systems being dependent on the RUB conjunction process. Another protein complex, the COP9-signaling complex (CSN), also regulates plant development, maturation, and cell differentiation [62]. The most prominent function of the CSN system is its role regarding the RUB enzyme isopeptidase, which repairs RUB modification by cullin proteins [63]. This process is dependent on CSN5, and any mutation in CSN5 leads to abnormal growth in plants [64,65]. Another protein complex, CAND1 (cullin-associated and neddylation-dissociated 1), was first isolated from animals and later functionally characterized in Arabidopsis through mutation approaches, suggesting its significant role in controlling the action of SCF systems [66,67]. A mutation in cand1 results in the malfunction of the responses of several phytohormones, such as gibberellic acid and auxin, to various stresses in Arabidopsis [68,69,70]. However, CAND1’s association with CUL1 enhances the activity of SCF systems [39,71]. Furthermore, CAND1 impacts both derubylation and rubylation after interactions within CRLs. The Skp1–F boxSkp2 and Rbx1 subunits are arranged and maintained more than 100 degrees apart due to Cul1’s stiff structure [72]. The cited studies proposed that the linking of CAND1 with the rotation of rubylation and derubylation overlaps with the calibration of the functioning of CRL systems [73,74].

**Figure 4:**
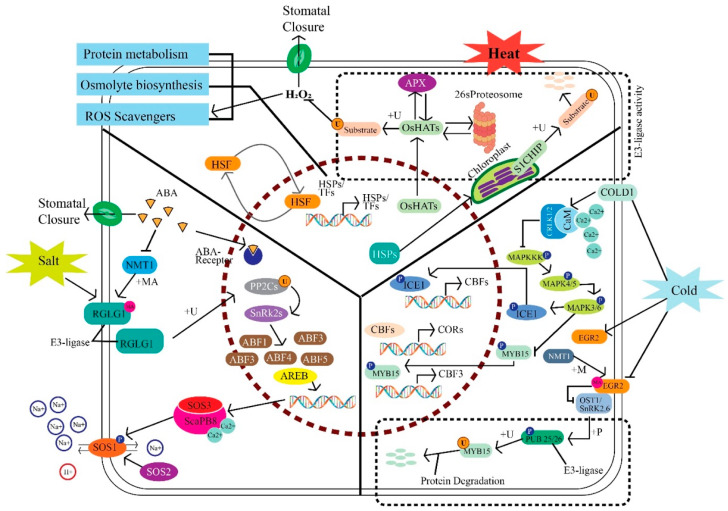
Graphical illustration of the molecular mechanism of cullin at the cellular level highlighting its role under different environmental stresses. The mechanism of protein degradation in the cytoplasm, the stress-response-mechanism-based upregulation of the gene interaction of proteins in the nucleus and the cytoplasm, and the significant role of cullin in hormone regulation are shown. Additionally, the pathways of osmolyte biosynthesis, ROS scavengers, and protein metabolism are demonstrated.

Proteolysis in the nucleus and cytoplasm is primarily mediated by the ubiquitin/26S proteasome pathway [73]. This route catalyzes the covalent binding of ubiquitin to substratum proteins in the E1–E2–E3 cascade [37]. The transfer of ubiquitin from E2 to a substrate is catalyzed by ubiquitin E3 ligases in interaction with substrates [75]. The interplay between heat and cold stress responses is further underscored by the shared involvement of E3 ligases in regulating protein degradation and gene expression. By mediating the ubiquitination and subsequent proteasomal degradation of unnecessary or damaged proteins, E3 ligases ensure that plants can efficiently respond to multiple abiotic stresses, highlighting their central role in maintaining cellular homeostasis and stress resilience (Figure 4).

## 4. Cullins’ Roles in Plant Growth and Development

### 4.1. Role in Cell Cycle Regulation

Plant cell growth and development are regulated by their physiological requirements and developmental stages [76]. In multicellular organisms, the cell cycle is composed of several phases: G1, S, G2, and M phases. Among these, the M phase is the most prominent phase regulating the cell cycle. Studies demonstrated that a group of proteins, including APC/C and, specifically, APC/C FZR/CCS52, is crucial for the initiation of an endoreplication cycle in plants because of this complex’s mitotic cyclin breakdown capacity [77,78]. However, another group of protein ligases called SCF (Skp-Cullin1-F-Box) was also revealed to play a significant role in the improvement of the cell cycle [79]. SCF complexes regulate the transformation of the G1 phase to the S phase through the degradation of cyclin-dependent kinase inhibitors (CKIs), and this process releases CDK, which is necessary for S-phase entry, wherein the E3 ubiquitin ligases are needed to degrade kinase inhibitors such as Sic1 and p27 Kip1 [80]. In addition to SCF, several other cullins such as CRLs (cullin–RING ubiquitin ligases) are also involved in this degeneration and cell cycle trolling phase. For instance, in Arabidopsis, there are over several hundred CRL receptor proteins, including BTB, F-box, and WD40 (DWD) [81]. According to data from the literature, out of five cullins (*CLU1-CLU5*), only three (*CUL1*, *CUL3A*, and *CUL4*) are expressed in immature trichomes [82,83]. Additionally, the importance of all three cullins for plant growth was validated using gene silencing and mutation approaches. Mutants lacking either *CUL3A* or *CUL1* showed no changes in the size or composition of heredity material [84]. However, determining the role of mutant cullin function during trichome development was challenging [41,84]. The small and weak trichomes were observed under the disruption of *CUL4* [85]. As a result, it was determined that the CUL4–CRL complex plays a major part in endoreplication regulation in trichomes, although the possibility that other CRLs and some types of E3 ligases are involved in the control of endoreplication cannot be completely ruled out [33].

### 4.2. Contribution of Cullin to Organ Development and Differentiation

Cullin plays an integral role in the development and differentiation mechanisms of plant tissues. It is involved in the regulation of the transcription factors of genes that are involved in cell division, cell cycle control, and, mainly, cell wall synthesis [76,86]. For instance, MADS-box (SKP1-Cullin-Fbox) controls phytohormone production and organ development, such as for copulatory organs, and floral meristem patterns are mediated by MADS-box genes such as *AGAMOUS* (*AG*) [75,87,88]. Additionally, another MADS-box gene, *FUL*, controls cell differentiation during fruit growth by impacting the fruit size and cell wall synthesis [1,89]. Similarly, other MADS-box genes such as *PFAG1* and *PFAG2* have also been identified as crucial factors in flowering and fruit maturation [90]. Furthermore, the HD–Zip transcription factors, regulated by cullin proteins, further influence carpel and leaf development adaptation, reviewed by [91]. Moreover, *CaCullin-1* (*Capana11g002239*) and *Cwf15/Cwc15* are implicated in controlling cell proliferation and cell wall synthesis from the ovary stage to fruit ripening in tall-fruited pepper plants [92]. Thus, cullin proteins significantly contribute to the differentiation of plant organs, exerting their effects through the regulation of key genes involved in various aspects of organ growth and patterning.

### 4.3. Influence of Cullin on Hormone Signaling Pathways

Cullin proteins play a vital role in plant hormone-signaling pathways, which are crucial for plants’ growth and development [56,71,93]. As cullin is a part of the ubiquitin–proteasome system, this assembled protein complex facilitates the selective degradation of key regulatory proteins, thereby regulating hormone responses [10,11,48]. For instance, in the auxin-signaling pathway, excess indole acetic acid interacts with the SCF^TIR/AFB–^Aux/IAA complex, promoting the ubiquitination of Aux/IAA proteins [94,95]. These ubiquitination-tagged Aux/IAA proteins are then degraded by the 26S proteasome, releasing ARF (auxin response factor), which activates the downstream gene expression to mediate auxin responses [96,97,98]. Several functional studies on the mutational effect of auxin receptors have provided primary information regarding auxin effector proteins such as AFB1, AFB2, AFB3, AFB4, AFB5, and TIR1 in Arabidopsis [99,100]. These mutant auxin protein receptors have different binding affinities for synthetic auxin-like quinclorac, and 2,4-dichlorophenoxyacetic acid has been found to have an affinity for TIR, while florpyrauxifen-benzyl preferentially binds to the AFB5 IAA co-receptor over the TIR1 receptor [101,102]. Due to the mutational effects that change the specificity of a receptor, the florpyrauxifen-benzyl effectively controls the growth of barnyard grass that has developed resistance to quinclorac (a widely used synthetic auxin herbicide).

Similarly, another hormone, gibberellin, plays a role in the regulation of various aspects of plant physiology by influencing transcriptional and post-transcriptional modifications at the cellular level, and cullin potentially plays a role in the regulation of this hormone [103,104]. The GA-signaling response also involves numerous complexes and protein interactions that maintain homeostasis. The key regulators include GA biosynthesis, GA effectors, and GA-deactivating enzymes [105]. Furthermore, the GA-and-Della-repressor interaction is very crucial for the regulation of plant growth and development. This interaction leads to the formation of the GID1–GA–DELLA complex, which is recognized by the SCFGID2/SLY1 complex, an E3 ubiquitin ligase comprising cullin-associated proteins [103]. This recognition allows for the polyubiquitylation of DELLA, leading to its degradation by the 26S proteasome, which activates the phytochrome-interacting factors (PIFs) and basic helix–loop–helix (bHLH), activating the transcription of GA-associated genes that ultimately activate plants’ defense mechanisms against environmental stresses [106]. Thus, by supplementing plants with GA3, greatly enhancing their height, biomass, antioxidant network, and photosynthesis under various external circumstances, and by modulating cullin-mediated pathways, we can further enhance the yield of crops.

## 5. Cullins’ Roles in Abiotic Stresses

### 5.1. Abiotic Stress Management by Cullins

Cullin plays a significant role in the regulation of plants’ responses to various abiotic stresses [75]. These proteins are involved in the ubiquitin–proteasome pathway, which controls the degradation of key regulatory proteins involved in stress signaling and tolerance mechanisms [107,108]. Plant diseases caused by a variety of microbial pathogens pose a major threat to plant survival and crop yield across the globe. In addition to the susceptibility of host plants and pathogen virulence, plant diseases are greatly influenced by environmental conditions such as temperature, humidity, and light [109,110]. Previous studies reported that cullin3 (CUL3)-based E3 ubiquitin ligase and HOS15, a substrate adaptor in the cullin-1-based E3 ubiquitin ligase, mediate NPR1 ubiquitination, reviewed in [111]. Studies investigating the expression of the *NPR1* target gene in *cul3a/b* and *hos15* mutant plants under varying humidity conditions revealed intriguing findings. Notably, the expression of *PR1* was significantly lower in *cul3a/b* mutants compared to *Col-0* plants under low humidity. Moreover, under similar conditions, the absence of *CUL3A/B* in the mutant plants led to the loss of high humidity’s suppressive effect on the *NPR1* target gene [112]. In contrast, high humidity still significantly suppressed *PR1* gene expression in the *hos15*-disrupted plants [113]. Under high humidity, RNAseq analysis revealed that the CUL3B transcript induced the downregulation of this gene [112]. Protein ubiquitination is carried out by the E1–E2–E3 cascade in eukaryotic cells [114].

To evaluate the action of genes that are relevant to rice F-box protein under environmentally pressurized conditions, microarray analysis was utilized on complete RNA extracted from rice sprouts treated with salt, dryness, and low temperatures [115]. The microarray data and real-time PCR outcomes of several representative genes differentially expressed under adephic pressure conditions revealed that numerous genes were upregulated, while some F-Box genes were downregulated, under salt, drought, and cold stresses. The function of the Arabidopsis F-box protein COI1 within protective reactions coupled with jasmonic acid (JA) is complicated [116]. It is a crucial factor of a SCF E3 Ub ligase that is expected to direct JA signaling repressors towards the proteome for degeneration. COI1 interacts with the histone deacetylase RPD3b and may control the expression of genes related to the JA response by modifying RPD3b’s and many proteins’ activities. It was recently discovered that COI1 is necessary for a notably high volume of JA and wound-injected gene actions [117]. It was also revealed that SON1, another F-box protein, controls innate immunity in a reductive manner, presumably through the Ub-proteasome route [118]. The closest related orthologs of Arabidopsis COI1 are the F-box proteins Os01g63420 and Os05g37690, which may play a parallel role in rice. But in abiotic stress situations, these proteins did not induce any discernible differences.

### 5.2. Cullin-Mediated Response in Abiotic Stress-Tolerance Mechanisms

Drought is the type of stress that affects a plant’s yield the most, and extensive research has been conducted on its signal passage. Plants have evolved a number of ways to detect water scarcity and withstand strain in times of drought in order to prevent growth abnormalities brought on by such stresses. The ABA-unbound and ABA-based routes make up the majority of the signaling routes associated with drought-related reactions. There are two primary types of ABA-reliant paths: one is regulated through bZIPs, i.e., ABA INSENSITIVE5/ABRE-binding components/ABRE-binding proteins (ABI5/ABF/AREB) and ABA-responsive elements (ABRE), and the other is controlled by MYC/MYB and MYC-/MYB identification sequences (MYCRS/MYBRS) [119]. The ABA-unbound routes make use of distinct regulatory networks in which the members of the AP2/ERF group, CBF/DREB1(C-REPEAT BINDING FACTOR/DRE-BINDING PROTEIN 1) and DREB2, function as trans-acting components, and the dehydration-reactive compounds/Crepeat (DRE/CRT) act as cis-acting compounds [120]. The majority of CRL receptor proteins that have been recognized as being associated with drought pressure signaling (such as DWA1; DWA2; DOR; EDL3; and BPMs) are part of the ABA-dependent drought pathway, but the CRL and ABA-unbound drought-response relationship is not understood. Finding CRL complexes that have the ability to alter the maintenance of CBF/DREB1 and/or DREB2 proteins would, therefore, be significant. The quantity of UV light that can penetrate the ozone layer has significantly increased recently. As a result, UV now poses a significant environmental risk to plants. Studies have examined the impact of the UV-B (280–320 nm) wavelength on the activity of CRL4 complexes. This results in moderation through DDB2 (DAMAGE-SPECIFIC DNA BINDING PROTEIN 2), CSAat1B, and COCKAYNE SYNDROME A-LIKE PROTEIN 1A [121]. The CUL4–DDB1 complex can be associated with CSAat1A and B when they form heterotetramers. This relationship is believed to be crucial to a plant’s defense against UV-B-induced DNA lysis [121]. Furthermore, CSAat1A and DDB2 are essential for UV-B-induced injury healing. RUP1 (REPRESSOR OF UV-B PHOTOMORPHOGENESIS 1) and RUP2 are probably downregulators of UV-B reactions but likely do so via direct contact with UVR8, an important UV-B signaling upregulated [122]. Despite the lack of proof linking RUP1 and RUP2 to CUL4-DDB1, it has been suggested that they may function as elements of CRL4 through their shared possession of the DWD domain.

## 6. Cullins’ Responses in Biotic Stress Management

### 6.1. Interactions Between Cullins and Pathogen Effectors

A diverse range of biotic stressors, such as insect herbivory and bacterial, viral, and fungal infections, pose constant challenges to plants. The defense pathways of plants against insects and pathogens have been shown to involve several F-box proteins [123]. COI1 is well known for its role in defense and wound-healing processes [124]. The COI1-suppressor *Cos1* mutation has been shown to be involved in the wound-healing process and plays an important role in the broader defense mechanisms of plants [125]. *Son1* was isolated via suppressor-screening experiments conducted on plants with mutations in nim1-1, the gene controlling systemic acquired resistance [126]. SON1 has been shown to contribute to pathogen stability, indicating that SON1-assisted defense operates independently of SAR-related genes. It is believed that SON1 negatively regulates defense responses associated with SAR via ubiquitination due to its role as an F-box protein [127]. Every kind of biological attack is met with both general and specific defenses that frequently overlap, and there is mounting evidence that the ubiquitin–proteasome system (UPS) is involved in these various protection strategies [128]. Multiple stages of plant defense may benefit from the controlled proteolysis of endogenous or pathogen-produced proteins. Plants can identify and react to a wide variety of likely pathogenic species via PAMPs (pathogen-associated molecular patterns) through their innate immunity [129]. For instance, chitin, a component of the fungal cell wall, and flagellin, a protein from bacteria, trigger various defense mechanisms, including callose deposition and oxidative surges, in a wide range of plant species [130]. But through gene-for-gene mechanisms, plants also possess enhanced capacities to react to certain diseases. A pathogen might develop a component, typically a protein or peptide, that uniquely facilitates its pathogenesis in particular host plants [131,132]. If plant protein develops, it neutralizes the pathogenic element via restricted cell death by means of a hypersensitive response (HR) [133]. U-box E3 ligases containing ARM repeats play a significant role in plant defense [134]. In Arabidopsis, tens of proteins, including PHOR1, contain two domains that are associated with gibberellin (GA)-signaling pathways [135]. U-box serves as the E2 interchange range in these proteins, while the ARM replicating the protein–protein interaction range may either directly or accidentally help in substrate identification [136]. A few U-box/ARM repeat E3 ligases have been isolated through gene screening [137]. Several U-box/ARM-repeat-containing proteins appear to play crucial roles in plant defense mechanisms, both through innate immunity and R-mediated pathways [59]. The Pep25 fungal peptide elicitor in parsley (*Petroselinum crispum*) causes the transcript PcCMPG1/ELI17 (Cys-Met-Pro-Gly1/Elicitor-activated gene) to increase quickly, producing a U-box/ARM protein [138]. The U-box/ARM proteins are transcriptionally highly expressed in response to pathogens, and tomato (SlCMPG1) was used to confirm which NtCMPG1 contained E3 ubiquitin ligase [139,140].

### 6.2. Cullin-Mediated Response to Biotic Stresses

In Arabidopsis and some other plants, SGT1b and SGT1a, which are programmed in many R-mediated routes, and SGT1b can attach to RAR1, which can interact with SCf E3 ligases [141,142,143,144]. This process also seems to facilitate SCF-mediated curtailment of the activity of E3 ligase substrates in sprouted yeast and Arabidopsis, potentially indicating different R-gene-linked routes [145]. However, it is not clear whether SGT1b promotes or inhibits substrate degeneration in plants’ defense routes. SGT1b seems to act against RPS5: HA amassment induces an increase in Rx:HA levels in Arabidopsis plants, but SGT1 in *N. Benthamian* causes the Rx:HA levels to drop sharply [146,147,148]. UPS can undoubtedly help with this crucial procedure, but advanced approaches to investigating this possible relationship are necessary. Proteasome-inhibitor treatment had no effect on RPS5:HA strength in two contrasting trials [149]. In contrast, most studies concentrate on the addition of whole-R-protein abasement rate changes in response to pathogen challenges or changed levels of RAR1, HSP90, or SGT1 [150]. But HSP90 and SGT1 have promising connections to the ubiquitin–proteosome system (UPS). The function of cullin-based E3 ligases in R-protein permanency and signaling is further proof that N-mediated gene–gene resistance to tobacco mosaic virus (TMV) is provided through the inhibition of SKP1, SGT1, or COP9 signalosome components [151,152]. RIN4 is a protein that binds to the RPM1 and RPS2 R proteins, which experience controlled breakdowns during pathogen attacks through the involvement of UPS in this process [153,154]. The AvrRpt2 protein of *P. syringe* functions as a cysteine protease that is capable of cleaving at two different locations inside the RIN4 protein [155]. A single cleft in the vicinity of the C-terminus releases the remaining RIN4 protein while leaving a little C-terminal anchor lodged in the plasma membrane. The RIN4 discharged from the membrane is swiftly destroyed in an unknown proteasome-based process, as the N-terminal part of the protein is only visible in soluble proportions treated with proteasome prohibitors. AvrRpt2-assisted cleavage at the second location in RIN4 revealed asparagine, a tertiary damaging remnant that may be identified based on the UPS corridor, a process known as the N-end rule [156,157]. Ubiquitylation through the N-end rule is dependent on the type of N-terminal remnant; for example, some amino acids can be identified through an E3 ligase directly at this site [158]. Then, an arginyl class is added (secondary deposits) or an amino class is removed, and then another arginyl group is added (tertiary deposits, such as asparagine and glutamine) [159]. The existence of asparagine at the N-terminus of cleaved RIN4 results in a change in the remainder’s placing through the N-end rule, leading to E3 ligase ubiquitylation and degeneration of the RIN4 protein. Due to that, substituting asparagine with a remnant that has not been identified through the N-end route (glycine) maintains an N-terminal RIN4-30 N(N11G): the GFP-supporter protein [160]. This method may not be exclusive to RIN4, since different RIN4-like proteins have similar AvrRpt2 cleavage zones.

### 6.3. Strategies for Manipulating Cullins to Enhance Plants’ Resistance to Pathogens

**Figure 5:**
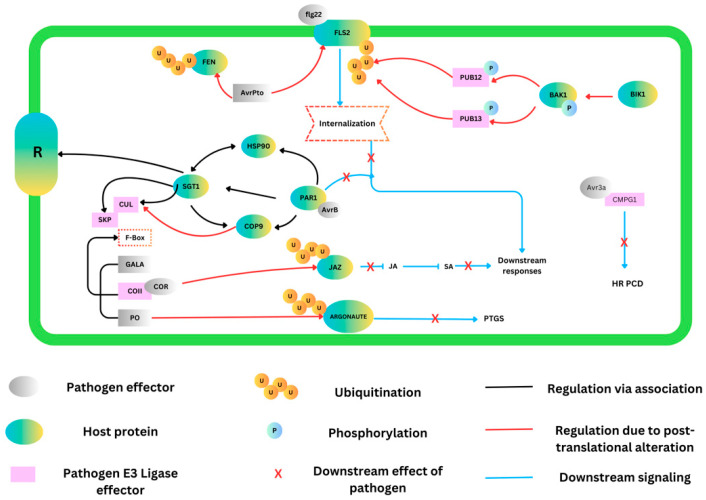
Pathogens target key components of the plant’s immune pathways, triggered by pathogen-associated molecular patterns (PAMPs) and effector recognition. For instance, the bacterial effector AvrPto interferes with the FLS2-mediated (flagellin perception) PAMP detection by exhibiting E3 ubiquitin ligase activity. Pathogen-encoded F-box motifs that are present in effectors such as GALA and PO bind to the host’s Skp1–Cullin–F-box (SCF) ubiquitin ligase complex, leading to the degradation of host factors such as ARGONAUTE, which blocks the post-transcriptional gene silencing of viral components. The bacterial toxin coronatine mimics active jasmonic acid (JA) and promotes the degradation of JAZ transcriptional repressors, activating the JA pathway and antagonizing the salicylic acid-mediated systemic acquired resistance (SAR) response. The bacterial effector AvrB targets the host protein RAR1, disrupting PAMP-triggered immune responses in susceptible interactions. Additionally, the oomycete effector Avr3a binds to and suppresses the CMPG1-mediated effector-triggered hypersensitive response and programmed cell death.

Cullin proteins, particularly through their role in the ubiquitin–proteasome pathway, are crucial for regulating protein degradation in plant cells, impacting a wide range of cellular processes, including responses to environmental cues and pathogens. By targeting cullins or modifying their downstream effects, researchers can enhance plants’ resistance to pathogens [161]. For instance, CRL1 (Cullin 1-RING E3 ubiquitin ligase) facilitates the ubiquitination and subsequent degradation of R receptor proteins, which are critical for preventing autoimmune reactions in plants [162]. Additionally, interactions involving proteins such as CSN5a/5b, which modulate the activity of cullins through Nedd8 removal, highlight the complex regulatory mechanisms governing cullin activity and plant immune responses [163]. The protein MVLG_05122, through its interaction with the CSN, could modulate the activity of CRL3, influencing the levels of NPR1, a key regulator of plants’ defense responses [164]. Genetic engineering approaches, such as overexpressing stress-responsive cullins or manipulating cullin–substrate interactions, have shown promise in enhancing the stability of proteins involved in pathogen resistance, thereby bolstering plant immunity [165,166]. Field studies in Arabidopsis species expression demonstrate the potential for these strategies to enhance resistance to pathogens. Pathogens target key components of the plant’s immune pathways, triggered by pathogen-associated molecular patterns (PAMPs) and effector recognition, which are shown in Figure 5.

## 7. Engineering Stress-Tolerant Crops Through Cullin Manipulation

Engineering stress-tolerant crops through targeted manipulation of cullins offers a powerful strategy for improving agricultural resilience [167]. By identifying and modifying stress-responsive cullins, scientists can enhance a plant’s ability to withstand environmental stresses such as drought, salinity, and extreme temperatures [168]. This can be achieved by overexpressing specific cullins or altering their interactions with substrates, thereby promoting the degradation of negative regulators or stabilizing proteins that confer stress tolerance [169]. Additionally, F-box proteins, which partner with cullins in the ubiquitination process, play significant roles in these responses. Although many F-box proteins are yet to be fully characterized, their potential in regulating stress responses in crops such as rice and Arabidopsis is substantial. Advanced techniques, including the modulation of cullin neddylation and CRISPR/Cas9 gene editing, allow for precise control over these pathways [170]. The integration of these approaches with chemical biology tools could synergistically enhance stress tolerance, leading to the development of high-yielding crops that are capable of thriving in challenging environments. However, successful application requires extensive field testing and validation to ensure that these traits are effective under real-world conditions. Collaborative efforts across scientific disciplines and careful consideration of biosafety regulations are essential for the successful implementation of cullin-based strategies in crop improvement.

## 8. Advanced Techniques in Cullin Research

### 8.1. High-Throughput Screening Methods for Identifying Cullin Substrates

High-throughput screening methods play a pivotal role in identifying cullin substrates, which are crucial for understanding the regulatory networks that are controlled by cullin–RING ligases (CRLs) and their roles in various cellular processes. A general method of establishing candidate E3 ligase substrates is to relate the accumulative genes in resistant and susceptible cells’ ligases [171,172]. Another way of amassing this contour is amplifying hexa–histidine-tagged ubiquitin (His_6_-Ub), tracked using Ni–NTA knockdown. This method involves the retrieval of polyubiquitinated peptides with Ni^2+^ ion similarity chromatography after adding refined His_6_-Ub to a wheat sprout [173]. It has been further revealed that His_6_-Ub can relocate resistant ubiquitin expression in yeast, and that His_6_-Ub that has been altered to stop polyubiquitin series creation can be expressed in Arabidopsis to refine the recapturing of ubiquitinated peptides [174]. The same method was applied to obtain the first SUMOylome in Arabidopsis, containing 357 assumed targets [175]. But the alteration or upregulation of ubiquitin may lead to unusual substratum ubiquitination. Yet another way depends on the immune precipitation of the distinctive diglycine deposit that remains linked to the ubiquitinated substratum lysine part following trypsin lysis. This enables the enhancement of ubiquitinated peptides without possible interference from the alteration of ubiquitin. Moreover, peptides altered by the ubiquitin-like peptides SUMO and RUB/NEDD8 also vacate the typical di-glycine part after trypsin degeneration [176].

### 8.2. Structural Biology Approaches to Elucidating Cullin–Protein Interactions

Structural biology approaches play a critical role in elucidating the intricate cullin–protein interactions that underpin the function of CRL complexes [177]. X-ray crystallography, nuclear magnetic resonance (NMR) spectrometry, and cryo-electron microscopy (cryo-EM) are key methods that are applied to envisage the 3D arrangements of cullins and their binding partners at the atomic scale [178]. The exemplary plant *Arabidopsis thaliana* has over 1600 genes involved in the ubiquitin–proteasome system (UPS), and 700 of these translate F-box peptides [11,179]. The three major subcomponents of the F-box in *A. thaliana* were identified based on the occurrence of precise domains such as F-box-associated domains (FBA-Ds), leucine-rich repeats, and kelch repeats [180]. The additional C-terminal spheres comprise WD-40, tetratricopeptide recurrences (TPRs), Tub, Armadillo (Arm), DEAD-like helicase, and jumonju (JmjC) [181]. Well-defined F-box DNA segments and their roles in plant growth are found across various plant species. FBA-D constitutes around 200 of the F-box peptides in *A. thaliana*, making it the main subset of F-box proteins [182]. The methods mentioned above provide a valuable understanding of the architecture of CRL complexes, the positioning of substrate receptors within cullin scaffolds, and the conformational changes that occur upon substrate binding or post-translational modifications [183]. X-ray crystallography allows researchers to obtain high-resolution structures of cullin domains and characterize their interactions with adaptor proteins and ubiquitin ligase components. NMR spectroscopy complements these studies by providing information on dynamic changes and protein dynamics in a solution, information that is particularly useful for studying flexible regions and transient interactions in cullin complexes [184]. Cryo-EM has emerged as a powerful tool for visualizing large macromolecular assemblies, enabling the visualization of intact CRL complexes and their interactions with substrate proteins in near-native states. Integrating structural biology data with biochemical and biophysical analyses enhances our understanding of cullin-mediated ubiquitination pathways, substrate recognition mechanisms, and the design of targeted interventions to modulate CRL activity for therapeutic applications for various diseases.

### 8.3. Genome-Wide Analysis of Cullin Gene Families in Plants

The genome-wide analysis of cullin gene families in plants provides a comprehensive understanding of the evolutionary and functional diversity of cullins across different species. Utilizing bioinformatics tools and genomic databases, researchers can identify and characterize cullin genes based on their sequence homology, domain organization, and phylogenetic relationships [24]. These analyses often reveal the presence of multiple cullin genes in plant genomes, organized into distinct subfamilies such as *CUL1*, *CUL3*, *CUL4*, and *CUL5* [25]. Genome-wide studies also uncover gene duplication events, intron–exon structures, and conserved protein motifs within cullin-coding sequences, providing insights into their evolutionary origins and functional specialization [24]. Furthermore, integrative analyses of cullin gene-expression patterns with respect to various developmental stages, environmental conditions, and stress responses contribute to our knowledge of their regulatory roles in plant growth, immunity, and adaptation [185]. Cullin peptides are variants of E3 ubiquitin ligase that are entwined in a range of physiological processes and resistances to stress in plants. In *Uncaria rhynchophylla*, the corresponding *CUL* gene subset has not been characterized, and its functions in plant growth, stress response, and natural product synthesis have not been explored. Twelve UrCUL gene associates, all containing the typical N-terminal domain and C-terminal domain and characterized based on *U. rhynchophylla* genetic data, were categorized into four subgroups based on their phylogenetic associations with CULs in *Arabidopsis thaliana* [186]. A genome-wide study in *Punica granatum* L. identified 56 U-box genes, classified into four groups. The study explored their chromosomal distribution, gene duplications, and expression profiles. It also highlighted that U-box genes are responsive to abiotic stress, predicting their functional roles [187]. The U-box gene family showed significant conservation of the U-box domain throughout this gene family. Duplicated genes allowed for the discernment of noticeable functional transitions among duplicated genes [188]. The gene-expression profiles of the U-box E3 family members show involvement in abiotic and biotic stress signaling, as well as hormonal pathways [189]. A genome-wide study in Brassica napus identified 200 ubiquitin-conjugating enzyme (UBC) genes, classified into 18 subgroups. Phylogenetic analysis revealed conserved gene architectures and motifs within subgroups, while gene-expression patterns and cis-acting regulatory elements indicated their potential roles in plant growth and development. Three BnUBCs were linked to traits influencing oil content and yield, providing valuable insights for future genetic research and breeding in B. napus [190]. The genomic information obtained from these studies serves as a valuable resource for designing targeted genetic engineering strategies, functional genomic experiments, and crop-improvement efforts aimed at enhancing plants’ resilience, productivity, and sustainability.

## 9. Applications of Cullin in Agriculture

Cullin proteins, particularly through their role in cullin–RING ligase complexes, hold significant potential for advancing agricultural practices by enabling the precise regulation of key physiological processes in crops [191]. These proteins are instrumental in modulating hormone-signaling pathways, thereby influencing growth, flowering, and stress responses. By fine-tuning the degradation of regulatory proteins through cullin-mediated pathways, researchers can optimize these traits to improve crop performance. Additionally, cullins are involved in plant defense mechanisms, where their manipulation can enhance resistance to pathogens and pests, reducing the need for chemical pesticides. Beyond defense, cullins also regulate nutrient uptake and homeostasis, impacting traits related to nutrient use efficiency, yield, and quality [111,192]. By integrating cullin-based approaches with advanced genetic engineering techniques, such as CRISPR/Cas9, there is a potential to create crops with tailored traits that enhance productivity, resource utilization, and environmental sustainability. These applications underscore the versatility of cullins as targets for genetic manipulation in the pursuit of more resilient and sustainable agricultural systems.

### Potential of Using Cullin-Based Biotechnological Interventions in Crop Improvement

Cullins hold immense potential for biotechnological interventions aimed at enhancing crop improvement through targeted genetic modifications. By manipulating cullin-mediated protein-degradation pathways, researchers can modulate key regulatory processes in plants, leading to improvements in various agronomic traits. One promising avenue is the engineering of stress-tolerant crops by targeting cullins that are involved in stress-response pathways [193,194]. For instance, the overexpression of stress-responsive cullins or the inhibition of cullins targeting stress-related proteins for degradation can enhance plants’ resilience to abiotic stresses such as drought, salinity, and extreme temperatures. Cullin-based interventions also offer opportunities for fine-tuning hormone-signaling pathways, leading to improvements in growth, flowering, and fruit-development characteristics. Moreover, manipulating cullins involved in plant defense mechanisms can result in crops with enhanced resistance to diseases and pests, reducing the need for chemical pesticides. The integration of cullin-based strategies with advanced biotechnological tools such as genome-editing technologies enables precise modifications of crop genomes, paving the way for the development of high-yielding, stress-tolerant, and environmentally sustainable crop varieties that are tailored to meet the challenges of modern agriculture [182]. Furthermore, cullins are integral components of hormone-signaling pathways in plants, controlling the abundance of key regulatory proteins involved in growth, development, and stress responses. By modulating cullin-mediated protein turnover, researchers can fine-tune hormone-signaling networks to improve traits such as plant architecture, flowering time, fruit development, and nutrient uptake [182]. For instance, targeted manipulation of cullins involved in auxin, cytokinin, or gibberellin signaling can lead to crops with enhanced growth characteristics, increased yields, and improved nutrient-use efficiency [195]. Similarly, engineering cullins associated with ethylene or abscisic acid pathways can confer drought tolerance or regulate fruit ripening, respectively, offering opportunities for crop enhancement and value addition.

Moreover, cullin-based interventions hold promise for enhancing plants’ defense mechanisms against pathogens and pests. Cullins participate in the degradation of defense-related proteins, including pathogen-recognition receptors (PRRs), defense hormones, and antimicrobial peptides. By modulating cullin activity, either via the overexpression, inhibition, or manipulation of substrate specificity, it is possible to enhance the accumulation of defense proteins and activate immune responses in plants [196]. This approach can lead to the development of crops with enhanced resistance to diseases and pests, reducing the need for chemical pesticides and promoting environmentally friendly agricultural practices [197]. The integration of cullin-based strategies, with advanced biotechnological tools, such as CRISPR/Cas9-mediated genome editing and miRNAs, offers precise and efficient ways of engineering crop genomes [198]. This integration enables researchers to make targeted modifications to specific cullin genes or regulatory elements, fine-tuning their expression or activity to achieve desired agronomic traits. Furthermore, leveraging high-throughput screening methods and computational approaches enhances the discovery and validation of cullin targets for crop improvement [199]. In summary, cullin-based biotechnological interventions present a powerful and versatile toolkit for crop improvement, encompassing stress-tolerance enhancement, hormone-signaling modulation, defense-mechanism activation, and targeted genome engineering. These interventions have the potential to contribute significantly to sustainable agriculture by creating high-yielding, stress-tolerant, and environmentally friendly crop varieties that are tailored to meet the evolving demands of global food security and agricultural sustainability.

## 10. Prospects and Future Challenges in Harnessing Cullins for Sustainable Agriculture

**Figure 6:**
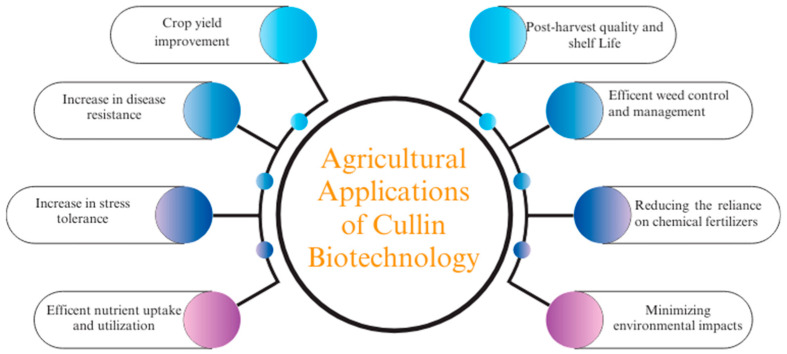
Overview of different applications of cullin-based biotechnology in agriculture.

The prospects of harnessing cullins for sustainable agriculture hold immense promise but also come with notable challenges that must be addressed. One of the key prospects lies in leveraging cullins to engineer crops with enhanced stress tolerance, improved nutrient-use efficiency, and heightened resilience to biotic and abiotic challenges. By precisely manipulating cullin-mediated protein-degradation pathways, researchers can target specific proteins involved in stress responses, hormone signaling, and defense mechanisms, leading to the development of high-performing crop varieties that are capable of thriving in diverse environmental conditions [200]. This approach aligns with the goals of sustainable agriculture by reducing our reliance on chemical inputs, increasing resource-use efficiency, and mitigating the impacts of climate change on crop productivity (Figure 6).

Furthermore, cullins offer opportunities for fine-tuning plants’ growth and development traits, such as their flowering time, fruit ripening, and biomass accumulation, through the targeted modulation of hormone-signaling pathways [201]. This opens avenues for improving the crop yield, quality, and market value while minimizing the corresponding environmental footprint [202,203,204,205]. Additionally, cullin-based interventions can contribute to the development of disease-resistant crops, reducing crop losses and promoting ecosystem health by minimizing the need for pesticides [206]. However, harnessing cullins for sustainable agriculture also poses several challenges that require careful consideration and innovative solutions. One challenge is the complexity of cullin-mediated protein-degradation networks, which involve multiple cullin isoforms, substrate receptors, and regulatory proteins. Understanding the specificity and dynamics of these interactions is crucial for designing effective genetic engineering strategies without causing unintended side effects or disrupting essential cellular processes [207]. Another challenge is ensuring regulatory approval and public acceptance of genetically modified crops that are engineered using cullin-based approaches. Addressing safety concerns, conducting thorough risk assessments, and transparently communicating the benefits and risks of these technologies are essential for fostering trust and regulatory compliance. Furthermore, scaling up cullin-based interventions from laboratory research to field application requires robust and cost-effective methods for genetically modifying crop plants. Developing efficient transformation protocols, optimizing gene-editing techniques, and addressing intellectual property rights and access issues are critical for facilitating technology transfer and adoption by farmers.

Collaboration across interdisciplinary fields, including molecular biology, genetics, agronomy, bioinformatics, and regulatory science, is essential for advancing the use of cullin-based strategies in sustainable agriculture. Embracing open-access data sharing, fostering international collaborations, and prioritizing ethical and socio-economic considerations can help us navigate through the challenges and unlock the full potential of harnessing cullins for creating resilient, productive, and environmentally sustainable agricultural systems.

### Ethical Considerations and Regulatory Aspects of Deploying Cullin-Based Technologies in the Field

Deploying cullin-based technologies in agriculture introduces important ethical considerations and regulatory challenges that must be carefully addressed. One such ethical consideration revolves around the potential environmental impact of genetically modified crops engineered using cullin-based approaches [23,208,209]. Assessing the ecological consequences, such as the gene flow to wild relatives, unintended effects on non-target organisms, and the disruption of natural ecosystems, is crucial for ensuring the sustainability and safety of these technologies [210,211]. Ethical discussions also encompass the equitable distribution of benefits and risks associated with cullin-based interventions, considering the needs and perspectives of diverse stakeholders, including farmers, consumers, and local communities. Moreover, regulatory aspects play a pivotal role in the oversight of the development, testing, and commercialization of cullin-based agricultural products. Regulatory frameworks vary globally, requiring thorough risk assessments, environmental impact assessments, and safety evaluations to obtain regulatory approval for genetically modified crops. Transparency, scientific rigor, and adherence to regulatory guidelines are paramount in ensuring the safety, efficacy, and ethical use of cullin-based technologies in agriculture. Additionally, intellectual property rights and access to genetic resources pose regulatory challenges in deploying cullin-based technologies. Balancing innovation incentives with fair access to genetic resources, and sharing benefits equitably among stakeholders, is essential for promoting innovation, fostering collaboration, and addressing socio-economic considerations.

Engaging in inclusive dialogs, stakeholder consultations, and public engagement activities is crucial for navigating the ethical and regulatory complexities associated with cullin-based technologies. Emphasizing ethical principles, such as transparency, accountability, precaution, and respect for biodiversity and human rights, can help guide the responsible deployment and governance of cullin-based interventions in agriculture. Collaborative efforts among governments, regulatory agencies, industry stakeholders, researchers, and civil society organizations are essential for developing robust regulatory frameworks, ethical guidelines, and best practices to ensure the responsible and sustainable use of cullin-based technologies for addressing global food security challenges while safeguarding environmental and societal well-being.

## 11. Conclusions

Cullins, integral players in plant biology, offer a multifaceted toolkit for agricultural innovation. Their structural intricacies and regulatory roles extend into pivotal areas such as growth modulation, stress responses, and immune defenses, laying a foundation for targeted interventions in crop improvement. Harnessing cullins enables the precision engineering of stress-tolerant crops that are resilient to biotic and abiotic challenges, heralding sustainable agriculture practices. However, the ethical dimensions and regulatory frameworks surrounding cullin-based technologies necessitate thoughtful considerations and transparent governance to ensure environmental and societal well-being. Moving forward, collaborative efforts, continued research, and the responsible deployment of cullin-centric strategies are imperative in navigating the complexities of agricultural sustainability and addressing global food security challenges effectively.
